# Circumferential (360°) trabeculotomy for steroid-induced glaucoma in adults

**DOI:** 10.1007/s00417-023-06012-5

**Published:** 2023-02-21

**Authors:** Laurentius J. (René) van Rijn, Catharina A. Eggink, Sarah F. Janssen

**Affiliations:** 1 Department of Ophthalmology, Amsterdam UMC, Location VUmc, PO Box 7057, 1007 MB, Amsterdam, The Netherlands; 2 Department of Ophthalmology, OLVG, Amsterdam, The Netherlands; 3 Department of Ophthalmology, Radboudumc, Nijmegen, The Netherlands

**Keywords:** Adult, Minimally invasive glaucoma surgery, Steroid glaucoma, Trabecular meshwork, Trabeculotomy

## Abstract

**Purpose:**

To evaluate the safety and efficacy of 360° circumferential trabeculotomy (TO) for steroid induced glaucoma (SIG) of short duration.

**Methods:**

Retrospective analysis of surgical results of 46 eyes of 35 patients undergoing microcatheter-assisted TO. All eyes had high intraocular pressure for at most about 3 years due to steroid use. Follow-up was between 2.63 and 47.9 months (mean 23.9, median 25.6).

**Results:**

Intraocular pressure (IOP) before surgery was 30.8 ± 8.3 mm Hg, with 3.8 ± 1.0 pressure-lowering medications. After 1 to 2 years, mean IOP was 11.2 ± 2.6 mm Hg (*n* = 28); mean number of IOP-lowering medications was 0.9 ± 1.3. At their last follow-up, 45 eyes had an IOP < 21 mm Hg, and 39 eyes had an IOP < 18 mm Hg with or without medication. After 2 years, the estimated probability of having an IOP below 18 mm Hg (with or without medication) was 85 ± 6%, and the estimated probability of not using medication was 56 ± 7%. Steroid response was no longer present in all eyes receiving steroids after surgery. Minor complications consisted of hyphema, transient hypotony, or hypertony. One eye proceeded to receiving a glaucoma drainage implant.

**Conclusion:**

TO is particularly effective in SIG with relative short duration. This concurs with the pathophysiology of the outflow system. This procedure seems particularly suited for eyes for which target pressures in the mid-teens are acceptable, particularly when chronic use of steroids is necessary .


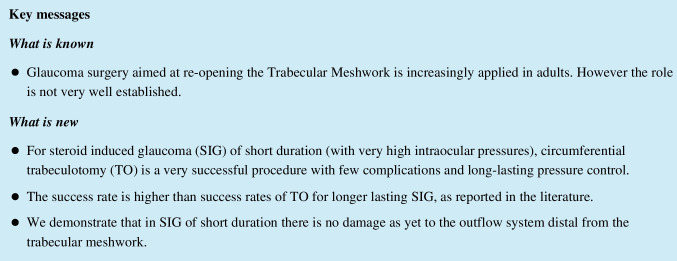


## Introduction

The main surgical treatment of glaucoma in adults consists of creating a bypass drainage. In trabeculectomy (TE) as well as in glaucoma drainage devices (GDD’s), the original outflow pathway via trabecular meshwork (TM), Schlemm’s canal, and collector channels is bypassed. Opening of the original drainage pathway by means of angle surgery (trabeculotomy (TO) or goniotomy) remains the main surgery for many forms of glaucoma in children [1]. There are some reports about successful angle surgery in specific conditions such as uveitic glaucoma and steroid-induced glaucoma (SIG).

In recent years, new techniques in glaucoma surgery were developed in the form of MIGS: minimal invasive glaucoma surgery. In general, two forms of MIGS may be distinguished: those that reopen Schlemm’s canal, such as iStent, Trabectome, and ab interno trabeculotomy and those that bypass the original outflow track such as PRESERFLO and XEN Gel Stent. Success of procedures that reopen Schlemm’s canal may depend on the integrity of the distal outflow pathways.

The physiology of the outflow track is complex and not completely understood. It is thought that in early open angle glaucoma, only the trabecular meshwork is obstructed whereas in later stages of the disease, a collapse of Schlemm’s canal occurs, in conjunction with a dysfunction of collector channels [2].

From this hypothesis follows that (MIGS) procedures that reopen the TM should work best in the form of open angle glaucoma with a short duration of existence. In primary open angle glaucoma (POAG), this duration cannot be determined reliably since the disease has a long preclinical asymptomatic phase. Therefore, surgery that reopens TM is probably unpredictable in POAG [3, 4].

In patients with SIG, the primary outflow resistance is thought to be located in the TM, through morphological and biochemical changes [5–7]. For SIG of short duration, at least a major part of the increased outflow resistance lies in the TM. For SIG of longer duration, just as in POAG, it cannot be ruled out that Schlemm’s canal and/or collector channels are also affected. Therefore, patients with recently developed SIG may benefit from angle surgery such as TO or goniotomy, other than patients with POAG or SIG with longer duration (and no IOP improvement after cessation of steroids). Iwao et al. demonstrated that TO works just as well as TE for SIG but has less complications [3]. The authors did not take the duration of existence of the SIG into account.

The present study comprises a retrospective analysis of our TO results in patients with SIG of short duration.

## Materials and methods

TO was offered to patients with a recently developed SIG, necessitating surgery. All eyes developed SIG because of necessary treatment with high doses of steroids due to their underlying eye disease (Table 1). Prior to the development of SIG, there had to be no signs of glaucoma: patients on chronic IOP lowering medication prior to the admission of steroids were excluded. All eyes that were included had a documented SIG of less than about 3 years. We consider a steroid response active when it was recently demonstrated that IOP rose when steroids were increased or commenced and/or that IOP dropped when steroids were decreased or stopped. On gonioscopy, the angle was largely open (at least Spaeth grade 3, scleral spur visible) and no or only minimal peripheral anterior synechiae were allowed.

**Table 1:** Characteristics of patients and eyes. “Intravitreal or subtenon,” “topical,” “systemic” refer to the application of the steroids

Diagnosis underlying steroid use	No. of eyes	No. of patients	No. of eyes: intravitreal or subtenon	No. of eyes: topical	No. of eyes: systemic	IOP 1mo before TO (mm Hg)	No. of PLMs 1mo before TO
Cornea	16	13	4	12	0	28.9 ± 6.5	3.3 ± 0.9
Uveitis	16	12	4	9	3	31.5 ± 10.0	3.5 ± 0.8
Diabetic macular edema	5	3	5	0	0	29.9 ± 12.9	4.8 ± 1.0
Cystoid macular edema after VR surgery	7	6	4	1	0	28.1 ± 7.3	3.6 ± 0.8
Systemic condition	2	1	0	0	2	37.5 ± 6.36	3.5 ± 0.7
Total	46	35	17	22	5	30.8 ± 8.3	3.8 ± 1.0

The IOP is expressed as mean ± SD (mm Hg). *PLMs*, number of pressure-lowering medications; *VR*, vitreoretinal

All patients needed surgery because of medically uncontrollable IOPs and/or necessity of chronic long-term steroid usage such as in corneal transplants or uveitis. When a patient was not suitable for TO, regular drainage glaucoma surgery was performed (usually Baerveldt GDD).

All patients received a diligent explanation of the nature of the procedure, expected results and possible alternatives, after which they gave their consent for the surgery. Because this study concerns a retrospective analysis of normal patient care, no formal approval of a Medical Ethical Committee was necessary (waiver obtained from the Medical Ethical Committee, Radboudumc Nijmegen). The study follows the guidelines of the Helsinki Declaration.

Ab externo microcatheter-assisted TO was performed either under general or subtenon anesthesia. A peritomy over 3 clock hours was made, followed by an external scleral flap of 3 × 3 mm. A triangular internal flap was created (1 × 3 mm) and Schlemm’s canal was deroofed. An illuminated microcatheter (Itrack, Ellex, Fremont, USA) was passed, mostly over 360°. The anterior chamber was filled with viscoelastic material. Both ends of the microcatheter were pulled such that the TM was cut and the catheter moved to the anterior chamber. The catheter was removed, viscoelastic material evacuated, and both flaps and the paracentesis were closed with Nylon 10-0. The conjunctiva was closed with Vicryl 8-0. A small air bubble was left in the anterior chamber. When 360° passage was not possible, the angle was opened over 180° using Harms trabecular probes (Geuder GmbH, Heidelberg, Germany) (one eye), or the catheter was picked up externally at the site of the stop via a small incision to create a partial trabeculotomy (two eyes, both 220°). Postoperative management consisted of topical antibiotics for 1 week and steroids, typically four times per day for at least 1 month.

**Fig. 1:**
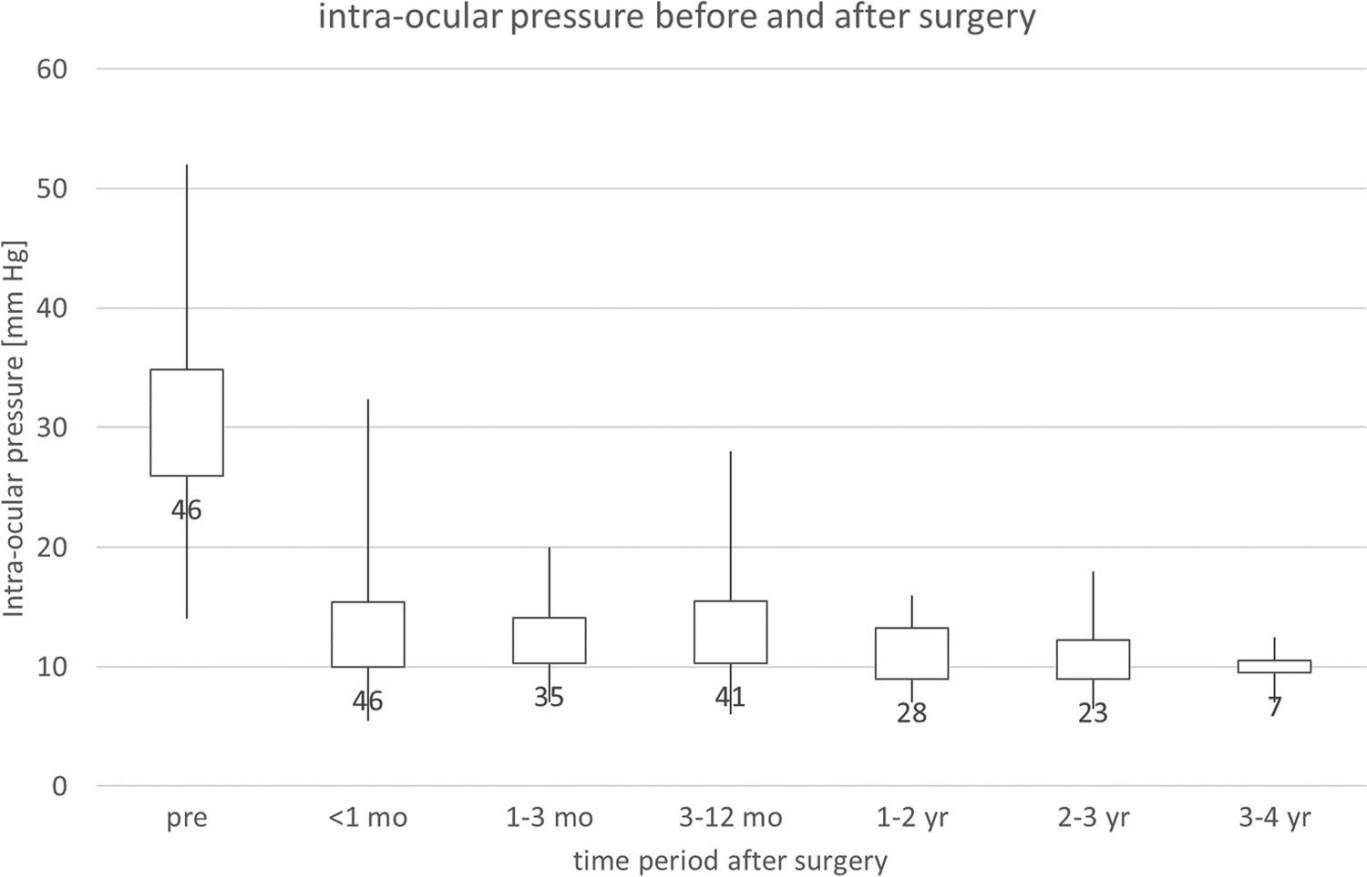
Intraocular pressures before and after surgery. *X*-axis: time intervals [days] starting at the indicated number and ending at the next number. *Y*-axis: intraocular pressure [mm Hg]. Box and whisker plots: data range [minimum, lower quartile, upper quartile, maximum]. Data label: number of eyes

**Fig. 2:**
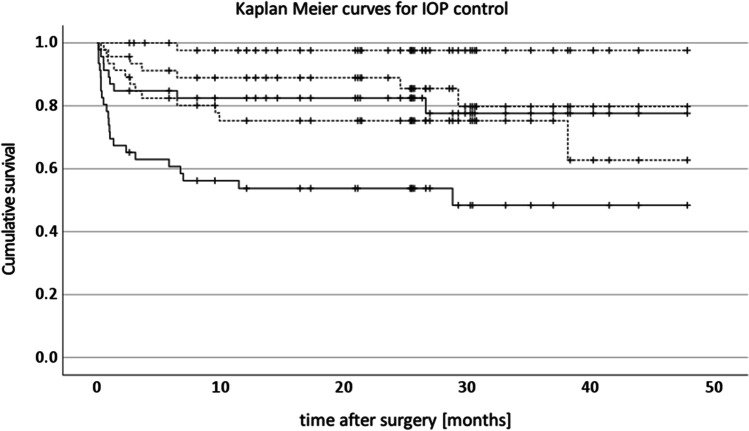
Kaplan–Meier curves for control of intra-ocular pressure after surgery. All curves represent the same eyes, with different criteria for failure. The dashed curves show the probability of having an IOP ≤ 21, ≤ 18 or ≤ 15 mm Hg, respectively, with or without IOP-lowering medication (qualified success). The solid curves show the probability of having an IOP of 18 and below or 15 and below, respectively, without IOP-lowering medication (complete success). All cases not using IOP-lowering medication had an IOP ≤ 18 mm Hg

Statistical analysis was performed using IBM SPSS statistics for PC version 27. Cox regression was used to investigate factors, determining success. Kaplan–Meier analysis was used to calculate survival and create the survival curve (Fig. 2). Figure 1 was created using Microsoft Excel with output from SPSS. The number pressure lowering medications (PLMs) before and after surgery: topical betablockers, prostaglandins, alpha-agonists, parasympathicomimetic agents and carboanhydrase inhibitors, and systemic acetazolamide were each counted as one.

## Results

### Patient descriptions

Forty-six eyes of 35 patients were included (mean age 46.6 ± 15.6 years, 51% male). Surgeries were performed between January 2017 and June 2021, in Amsterdam by LJR and in Nijmegen by CAE.

The conditions necessitating steroids were related to the cornea, uveitis, diabetic macular edema, persistent edema after vitreoretinal surgery, and systemic illness. SIG caused by topical, intravitreal, subtenon, and systemic steroids were included. The first high IOP (> 21 mm Hg) was noted 8.3 ± 10.6 (mean ± SD) months before surgery (median 3.5, range 0–40 months, *n* = 44). The first very high IOP (> 30 mm Hg) was noted 6.1 ± 10.6 (mean ± SD) months before surgery (median 1.0, range 0–40, *n* = 41). Mean IOP before surgery was 30.8 ± 8.3 mm Hg. (Table 1).

### Intraocular pressure and medication

Figure 1 shows the intraocular pressure before and after surgery. The median pressure was lower than 15 mm Hg throughout the follow-up period, and IOPs above 20 mm Hg were only found in the first month and in one patient after 3–12 months. This patient went on to receiving a GDD. The mean IOP was 30.8 ± 8.3 mm Hg before surgery, 13.3 ± 4.5 mm Hg after 3–12 months, and 11.2 ± 2.6 mm Hg after 1–2 years. Results after more than 2 years were similar, though the number of eyes was smaller.

Right before surgery, the number of PLMs was high: several patients used apraclonidine and acetazolamide above the regular glaucoma medication. The mean number of PLMs before surgery was 3.8 ± 1.0 mm Hg. The mean number of PLMs was 1.0 ± 1.3 Hg after 1–3 months, 1.0 ± 1.4 Hg after 3–12 months, and 0.9 ± 1.3 mm after 1–2 years. Results after more than 2 years were similar, though the number of eyes was smaller.

Figure 2 shows the Kaplan–Meier curve for IOP control with and without medication [8]. After surgery, except for one case, IOPs were lower than 21 mm Hg with medication. (Estimated survival 98 ± 2%, mean ± standard error.) Most cases needed medication to control their pressure after surgery. (Estimated probability of not using IOP-lowering medication: 39 ± 11%.) The one case with uncontrolled pressures was later well controlled after implantation of a GDD. Estimated 80 ± 8% of cases had an IOP (with medication) below 18 mm Hg, and estimated 61 ± 12% had an IOP (with medication) below 15 mm Hg.

### Factors determining success

By means of a Cox proportional hazard model, we investigated the role of several factors determining successful outcome. As dependent variable, we choose “IOP < 21 mm Hg without medication.” This variable provided better contrast than “IOP < 21 mm Hg” since the majority of eyes fulfilled the latter criterion. We investigated the factors such as diagnosis, mean IOP 1 month before surgery, hospital, and application of steroid (subtenon or intravitreal/topical/systemic). In a multivariate model and in successive univariate models, none of these factors was significant.

### Steroid use before and after surgery

All eyes received topical steroids during at least 4 weeks after surgery. Due to the underlying condition, in many cases, steroids needed to be continued after surgery. At 3–12 months, 28 of 39 eyes received some form of steroid therapy. We did not find a relation between discontinuation of steroids and success of surgery, indicating that the steroid response was no longer present. Ten eyes continued to receive either subtenon or intravitreal steroids. In all eyes, the IOP remained low and not significantly different from the eyes receiving none or only topical steroids.

### Complications

**Fig. 3:**
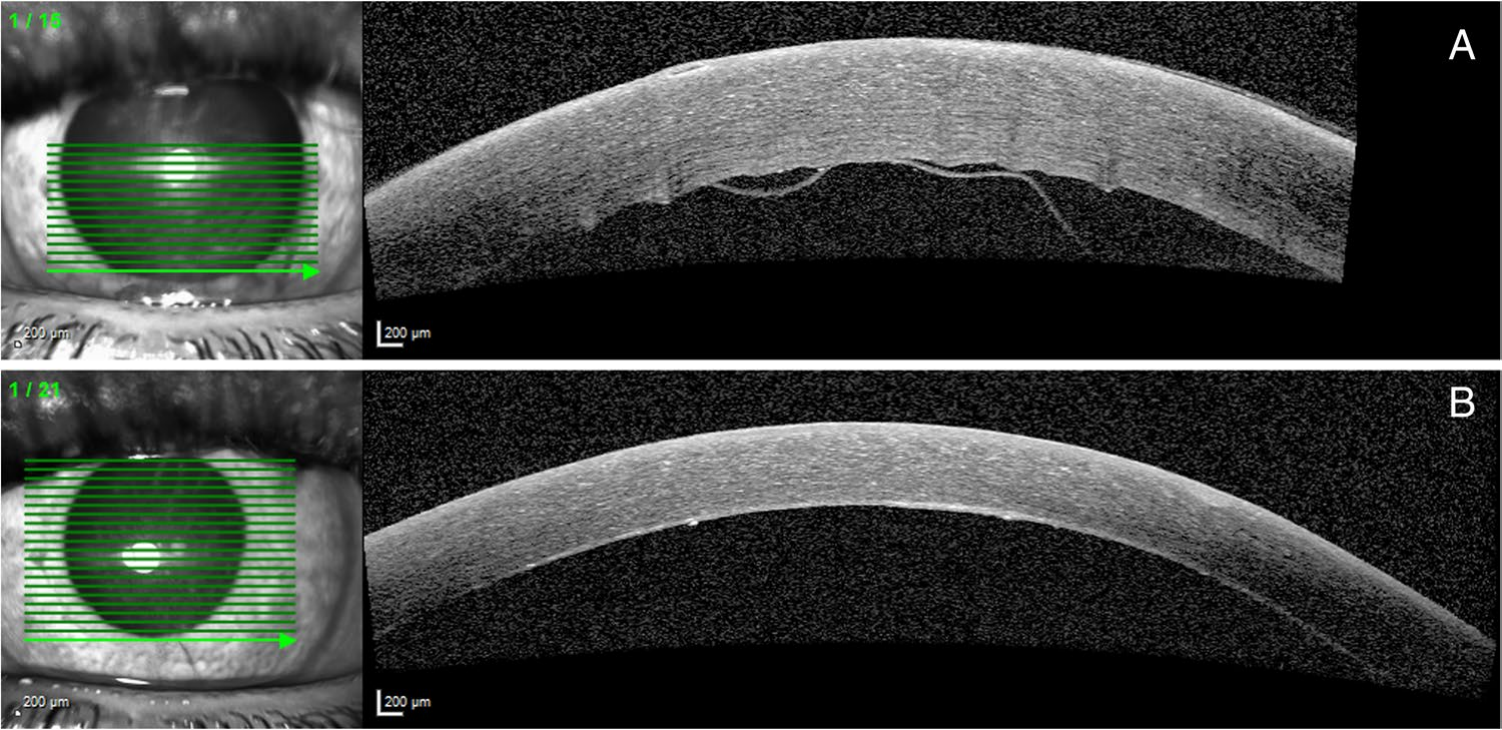
Detachment of Descemet’s membrane in one patient with spontaneous re-attachment (Heidelberg Spectralis anterior segment OCT). Panel **A** 3 days after surgery. Panel **B** 13 days after surgery

Complications consisted of transient hyphema (*n* = 14), postoperative hypotony (IOP below 5 mm Hg, *n* = 3), and postoperative hypertony (IOP above 21 mm Hg within 1 week after surgery, *n* = 6). All of those resolved within several days to weeks. For postoperative hypertony with hyphema, in two eyes, an anterior washout was performed and in one eye a paracentesis. In one patient, during the pulling of the catheter, a Descemet detachment was created. Upon noticing this, the pulling was stopped, and the catheter was repositioned after which the procedure could be continued. At the end of the surgery, a large air bubble was left in the anterior chamber. After surgery, there was a Descemet detachment over the lower cornea, which resolved spontaneously within 2 weeks (Fig. 3).

## Discussion

Our study demonstrates that ab externo TO is a good technique for SIG with a limited duration of existence. The intraocular pressure is well controlled after the surgery, and complications and burden to the patient are limited. Because very low IOPs or low IOPs without PLMs are often not achieved, this procedure seems particularly suited for eyes with, at most, moderately low target pressures; hence, eye with short lasting very high IOPs for which target pressures in the mid-teens are acceptable. TO seems particularly suited for eyes in continued need of treatment with steroids.

### Trabeculotomy for steroid induced glaucoma

Our findings are in accordance with other studies. Honjo et al. [9] performed TO in 14 eyes of seven patients with SIG. Initial pressures were high (34.6 ± 8.4 mm Hg); six patients were not able to stop their steroids. Their success rate was 83.6% (pressure lower than 21 mm Hg with medication) after 5 years. Iwao et al. [3], in a multicenter study, demonstrated that (120°) TO for SIG was much more successful than for POAG (73.5% vs 51.7% success (IOP < 22 mm Hg with or without medication) after 5 years). For SIG, TO was just as successful (73.5% vs 74.5% IOP < 22 mm Hg after 5 years) or less successful (51.7% vs 71.6% IOP < 19 mm Hg after 5 years) than TE. Complications were less in the TO than TE groups. Hence, TO was better suited for SIG than for POAG and, although successful, IOPs are somewhat higher after TO than after TE for SIG.

Other than our study, these studies did not distinguish between complete and qualified success and did not have a cut-off value for the duration of existence of SIG, as was commented on the paper by Iwao et al. [10, 11]. We speculate that our higher success rate (98% success if defined as IOP < 22 with or without medication) is due to our strict inclusion criterion: only SIG with limited duration of existence was included.

GATT was performed in 13 eyes with SIG, all of which had to continue using steroids after surgery [12]. Half of the patients had uveitis. The procedure was successful in all eyes with a mean IOP at 24 months of 10.5 mm Hg and an average number of medications of less than 1. Just as in our study, pressures and number of medications before surgery were high.

A higher preoperative IOP seems a poor prognostic factor for TO in POAG and exfoliative glaucoma [13], but not for TO in SIG [3]. Postoperative pressure spikes are frequently observed and do not hinder long-term success of the procedure [14].

Trabectome was successful for 20 eyes SIG [15], resulting in an IOP reduction from 33.8 to 15.0 mm Hg on average at 12 months after surgery. In a comparative study [16], it was reported that Trabectome-assisted goniotomy resulted in more pressure reduction in SIG than in POAG although a 12-month survival rate was similar at 85%.

### Trabeculotomy for other forms of glaucoma

Bao et al. [4], in a retrospective study, showed that TO had a lesser long-term success than TE for POAG in 25 and 20 eyes, respectively. The mean follow-up was 8 years.

### Selective laser trabeculoplasty for SIG

Recently, several authors reported about the efficacy of SLT for SIG. Zhou et al. [17] performed SLT in 29 eyes with SIG, with a mean baseline pressure of 27.6 mm Hg. They found 54% failure after 2 years (less than 20% IOP reduction more than 3 weeks after the SLT). This was similar to a subgroup of POAG eyes with comparable baseline IOPs. Generally, the IOP decreased 3–8 weeks after the SLT treatment. The rate of hypotensive medication reduced from 3.5 at baseline to 1.9 by 18 months AlObaida [18], applying SLT in 25 eyes with SIG, found an IOP reduction from 23.7 to 14.4 mm Hg on average 19 months after the treatment (39% reduction). This is similar to the pressure reduction found for early POAG and ocular hypertension in the LIGHT trial [19]. Xiao et al. reported a gradual IOP reduction from 30.6 to 15.1 mm Hg (48%) at 12 months after the treatment in 16 eyes with SIG and quiescent uveitis. Four patients went on to surgery, two of which had anterior synechiae, potentially sequalae of an SLT. Maleki et al. [20] applied SLT in 15 eyes with SIG after a fluocinolone acetonide implant for uveitis. Seven eyes reached target with a pressure of 15.14 mm Hg (50.4% reduction) at 12 months after the treatment. Eight eyes failed after treatment within 12 months. The aforementioned studies demonstrate the effectiveness of SLT for SIG. Especially in SIG patients where the IOP is not extremely high and/or there is time to wait for the effect of SLT (on average 6 weeks), SLT might be an elegant first step in the attempt to lower the IOP in SIG. However, when the IOP is too high, one may directly choose for surgery, as we did with our patients.

### Mechanics of outflow path and pathophysiology of glaucoma

The primary regulation of IOP resides in the outflow system [21, 22]. Removal of TM resulted in a reduction of 75% in outflow resistance in normal eyes and removal of abnormal resistance in glaucomatous eyes [22]. Schlemm’s canal lumen decreases at higher levels of IOP [23]. Remarkably, removal of the outer wall also resulted in a 75% reduction of outflow resistance [24].

Kagemann et al. [25] showed in an in vitro OCT study that cross-sectional Schlemm’s canal surface was smaller in glaucoma patients than in normal controls. Increase of episcleral venous pressure results in increase of Schlemm’s canal diameter [26]. Apposition of the TM with Schlemm’s canal outer wall results in an increase in outflow resistance, a mechanism that can be eliminated by keeping the TM away from Schlemm’s canal outer wall [27] or by TO [24]. Outflow resistance is higher at high intraocular pressures than at low pressures, presumably through collapse of Schlemm’s canal and entrance of the TM in the collector channel entrances [28].

Contractile elements within the wall of Schlemm’s canal seem to modulate flow into the collector channels [29, 30]. This only seems to work when IOP is in the normal range [31]. Increasing episcleral venous pressure causes reflux of blood into Schlemm’s canal, indicating patency of the deep scleral plexus/collector channels. This reflux fails progressively with advancement of glaucoma [32, 33]. The anatomy and physiology of the outflow pathway have been comprehensively reviewed [2]. Consequences for (limitations of) MIG procedures have been discussed [34, 35].

It may be anticipated that both removal of SC inner wall and SC outer wall may reduce outflow resistance. Removal of SC outer wall (sinusotomy, deep sclerectomy) may reduce IOP. Success of stretching of Schlemm’s canal inner wall (canaloplasty) seems to depend on the patency of the distal outflow system. Grieshaber et al. [36, 37] demonstrated that blood reflux is associated with a higher success rate of the procedure. Bleaching of episcleral vessels after Trabectome is associated with success of the procedure [38, 39]. In cases without patent distal outflow system, there may be damage to the distal collector channels [25, 40].

SLT reduces IOP by about 20% [41, 42], but it seems more effective in newly diagnosed glaucoma [31], compliant with the hypothesis of a relatively intact distal outflow system early after diagnosis.

### Limitations of the study

Limitations of our study comprise its retrospective nature and absence of control groups. Some of our patients have uveitis; it is known that angle surgery works in uveitis as this may explain part of the effect of this surgery. From a theoretical point of view, we limited our procedure for those cases with SIG with a limited duration of existence or with “active” steroid response. From the present study, it cannot definitely be concluded whether TO works (or not) in SIG with a longer duration of existence.

## Conclusion

Our study demonstrates that TO is a valuable therapeutic option in the eyes with SIG of limited duration and with at most moderate glaucomatous damage. TO is less invasive than GDD surgery, and the pressure-lowering effect of TO seems to be long-lasting. Moreover, TO eliminates steroid-induced pressure rise. Since pressures in the low teens (without medication) are often not reached, care should be taken to apply TO in eyes with very low target pressures.
